# Editorial: High-impact respiratory RNA virus diseases

**DOI:** 10.3389/fvets.2023.1273650

**Published:** 2023-08-22

**Authors:** Victor Manuel Petrone-García, Inkar Castellanos-Huerta, Guillermo Tellez-Isaias

**Affiliations:** ^1^Departamento de Ciencias Pecuarias, Facultad de Estudios Superiores Cuautitlán UNAM, Cuautitlán Izcalli, Mexico; ^2^Department of Poultry Science, University of Arkansas, Fayetteville, AR, United States

**Keywords:** RNA viruses, mutation rate, COVID-19, PRRS, influenza, Newcastle diseases, canine distemper, African horse sickness

The text discusses the serious threat of high-impact “respiratory RNA virus” (RRV) infections to global health (1, 2). These viruses can spread rapidly, causing severe respiratory illnesses and significant socioeconomic burdens (3). The emergence and reemergence of these viruses continuously hinder public health preparedness and control measures (1). “RRVs” form a diverse group sharing genetic material of single and double-stranded RNA (4–6). They can lead to various respiratory disorders, from mild symptoms to life-threatening respiratory distress (5).

Notable examples of RRVs include canine distemper virus (CDV) (7) and Newcastle diseases virus (NDV) (8), Influenza virus (IV) (9), porcine reproductive and respiratory syndrome virus (PRRSV) (10), the coronavirus disease 2019 (COVID-19) caused by SARS-CoV-2 (11, 12), and the less well-known African horse sickness virus (AHSV) (13) are some of the most notable high-impact RRVs (14). Past pandemics, like the IV in humans and animals (15, 16) and SARS in the twenty-first century highlighted the challenges of zoonotic transmission and controlling outbreaks (11). The ongoing COVID-19 pandemic demonstrates the relevance of these diseases (17–21) (Jarrah et al.). RRVs can have substantial economic consequences, including disruptions to global trade, tourism, and healthcare systems (22–24). The interconnectedness of global health security underscores the importance of international collaboration, data sharing, and robust surveillance systems to detect and respond to emerging viral threats (25).

RRVs have the potential to spread widely, causing severe respiratory illnesses and far-reaching consequences beyond health, including economic disruptions and impacts on global trade, tourism, and healthcare systems (26). The emergence and reemergence of these viruses continuously challenge public health measures (1). RRVs refer to a diverse group of viruses with RNA genetic material, typically with a protein coat or capsid, surrounding their genome (27), causing various respiratory disorders in human and animal populations. Some RRVs have an envelope derived from the host cell membrane (28). The RNA virus-genomic structure and replication strategies can vary, influencing their ability to infect and replicate within host cells (29). RRVs are characterized by high mutation rates, rapid replication cycles, and the ability to cause various clinical consequences (5, 25, 30). Their genetic structure allows for (+) sense or (–) sense RNA, leading to diverse viral populations (31–33). RRVs reflex a higher mutation rate, enabling them to adapt and evolve rapidly, evading host immune responses and developing resistance to antiviral drugs (25, 34–36). The epidemiology of RRVs varies based on factors such as transmission mode, viral stability, host range, and population susceptibility (19, 37, 38). Some RRVs exhibit seasonal patterns, while others cause sporadic outbreaks or persistent endemic infections (39). The transmission dynamics depend on the specific virus and can occur through respiratory droplets, direct contact, vector-borne transmission, or fecal-oral transmission (19).

Diagnosing RRV diseases involves various laboratory techniques, including molecular methods like polymerase chain reaction (PCR) (40–43) (Machado et al.), and serological tests to detect viral genetic material or antibodies (ELISA test) (44–48). Overall, understanding the characteristics, transmission, and diagnostic methods of RRVs is crucial for effectively combating these infections and improving global health preparedness. Some examples of RRVs are briefly described below:

a) SARS-CoV-2 has a single-stranded (+) sense RNA virus genome, which means it can serve as a messenger RNA (mRNA) for protein synthesis once it enters a host cell (9, 49). The genome structure includes regions encoding structural and non-structural proteins (50, 51). Its genome can undergo mutations, leading to the emergence of different variants that may impact transmissibility, virulence, and vaccine efficacy (52–54). Continuous monitoring of viral genomic sequences helps researchers understand the evolution and spread of the virus (18, 23, 55) (Padilla-Blanco et al.). Diagnosing in animals is crucial for understanding its impact on animal health and welfare, studying transmission dynamics, adopting a One Health approach that considers human, animal, and environmental health, and enhancing public health and epidemiological surveillance (56). Animals can become infected with SARS-CoV-2 through zoonotic transmission, and studying such cases provides valuable data for tracking the virus's spread and assessing its potential impact on human populations. Collaborative efforts between human health professionals and veterinary experts are essential for comprehensive disease surveillance, prevention, and control (18, 57).
b) PRRSV is a significant viral pathogen affecting pigs worldwide (58). It is an enveloped, single-stranded RNA virus, family *Arteriviridae*, genus *Betaarterivirus* (59), with a genome containing several ORFs encoding viral proteins, including structural and non-structural proteins (60–62). There are two major genotypes: PRRSV type 1 (PRRSV-1) and PRRSV type 2 (PRRSV-2) (63). PRRSV is characterized by causing reproductive failure, respiratory illness, and immunosuppression in pigs (64). Its genetic diversity challenges disease control and vaccine efficacy (65, 66). Recently a new type of active vaccine demonstrated superior results against the PRRSV (Trevisan et al.).
c) IV is an enveloped RRV of the *Orthomyxoviridae* family classified into four genera: *Alphainfluenzavirus* (influenza A virus, IAV), *Betainfluenzavirus* (influenza B virus, IBV), *Gammainfluenzavirus* (influenza C virus, ICV), and *Deltainfluenzavirus* (influenza D virus, IDV) (67–69). The viral genome comprises segments of single-stranded RNA, encoding structural and non-structural proteins (68, 70). The replication and transcription processes involve interactions between viral components and host cell machinery (71). Influenza viruses can cause seasonal flu outbreaks (Fujiwara et al.), occasionally leading to pandemics (15, 16).
d) NDV is an enveloped, single-stranded RNA virus belonging to the *Avulavirus* genus in the *Paramyxoviridae* family (72). It affects domestic poultry and wild bird species (72). NDV has a non-segmented RNA genome, and its replication and transcription processes involve interactions between the viral polymerase complex and viral RNA (73, 74). Vaccination is crucial for controlling NDV outbreaks (8).
e) CDV is a highly contagious viral disease affecting dogs and other *Canidae* family members (75, 76). It belongs to the *Morbillivirus* genus within the *Paramyxoviridae* family (77). The CDV genome is segmented RNA and encodes various structural and non-structural proteins (78). Vaccination is the most effective preventive measure against CDV (79, 80).
f) AHS, a member of the *Orbivirus* genus within the *Reoviridae* family (13, 81), has a segmented genome with 10 RNA segments, each encoding specific proteins (82, 83). The virus is primarily transmitted through insect vectors, and vaccination and vector control are essential for disease prevention (82). AHS can cause various clinical signs, varying from peracute to chronic (Adesola et al.). Research focuses on understanding the viral genome and developing effective control strategies (83).

The challenges of prevention, diagnosis, and treatment are examined, highlighting the necessity for continuous research, surveillance, and preparedness. To mitigate the effects of RRVs, it is essential to prioritize surveillance, prevention, diagnostics, and research efforts while fostering collaboration among scientists, healthcare professionals, and policymakers to enhance global preparedness for future outbreaks.

## Author contributions

VP-G: Conceptualization, Formal analysis, Investigation, Writing—original draft, Writing—review and editing, Data curation, Methodology, Supervision. IC-H: Investigation, Writing—original draft, Writing—review and editing, Software, Visualization. GT-I: Funding acquisition, Supervision, Writing—review and editing, Project administration, Resources, Validation.

## References

[1] Tang JW Lam TT Zaraket H Lipkin WI Drews SJ Hatchette TF. Global epidemiology of non-influenza RNA respiratory viruses: data gaps and a growing need for surveillance. Lancet Infect Dis. (2017) 17:e320–6. 10.1016/S1473-3099(17)30238-4 28457597 PMC7164797

[2] Carrasco-Hernandez R Jácome R López Vidal Y Ponce de León S. Are RNA viruses candidate agents for the next global pandemic? A review. ILAR J. (2017) 58:343–58. 10.1093/ilar/ilx026 28985316 PMC7108571

[3] Alvarez-Munoz S Upegui-Porras N Gomez AP. Ramirez-Nieto G. Key factors that enable the pandemic potential of RNA viruses and inter-species transmission: a systematic review. Viruses. (2021) 13:537. 10.3390/v13040537 33804942 PMC8063802

[4] Chepur S Pluzhnikov N Chubar O Bakulina L Litvinenko I Makarov V. Respiratory RNA viruses: how to be prepared for an encounter with new pandemic virus strains. Biol Bull Rev. (2021) 11:154–71. 10.1134/S207908642102002X

[5] Kesson AM. Respiratory virus infections. Paediatr Respir Rev. (2007) 8:240–8. 10.1016/j.prrv.2007.07.003 17868922 PMC7106180

[6] Bremer C Huismans H Van Dijk A. Characterization and cloning of the African horsesickness virus genome. J Gen Virol. (1990) 71:793–9. 10.1099/0022-1317-71-4-793 2324709

[7] Deem SL Spelman LH Yates RA Montali RJ. Canine distemper in terrestrial carnivores: a review. J Zoo Wildl Med. (2000) 31:441–51. 10.1638/1042-7260(2000)031(0441:CDITCA)2.0.CO;2 11428391

[8] Wakamatsu N King DJ Seal BS Samal SK Brown CC. The pathogenesis of newcastle disease: a comparison of selected newcastle disease virus wild-type strains and their infectious clones. Virology. (2006) 353:333–43. 10.1016/j.virol.2006.06.013 16860365

[9] Javanian M Barary M Ghebrehewet S Koppolu V Vasigala V Ebrahimpour S. Brief review of influenza virus infection. J Med Virol. (2021) 93:4638–46. 10.1002/jmv.26990 33792930

[10] Cho JG Dee SA. Porcine reproductive and respiratory syndrome virus. Theriogenology. (2006) 66:655–62. 10.1016/j.theriogenology.2006.04.024 16730057

[11] Harapan H Itoh N Yufika A Winardi W Keam S Te H. Coronavirus disease (2019). (COVID-19): a literature review. J Infect Public Health. (2020) 13:667–73. 10.1016/j.jiph.2020.03.019 32340833 PMC7142680

[12] Gerges Harb J Noureldine HA Chedid G Eldine MN Abdallah DA Chedid NF. SARS MERS and COVID-19: clinical manifestations and organ-system complications: a mini review. Pathog Dis. (2020) 78: ftaa033. 10.1093/femspd/ftaa033 32633327 PMC7454523

[13] Carpenter S Mellor PS Fall AG Garros C Venter GJ. African horse sickness virus: history, transmission, current status. Annu Rev Entomol. (2017) 62:343–58. 10.1146/annurev-ento-031616-035010 28141961

[14] Lai CC Shih TP Ko WC Tang HJ Hsueh PR. Severe acute respiratory syndrome coronavirus 2 (SARS-CoV-2) and coronavirus disease-2019 (COVID-19): the epidemic and the challenges. Int J Antimicrob Agents. (2020) 55:105924. 10.1016/j.ijantimicag.2020.105924 32081636 PMC7127800

[15] Kilbourne ED. Influenza pandemics of the 20th century. Emerg Infect Dis. (2006) 12:9. 10.3201/eid1201.051254 16494710 PMC3291411

[16] Capua I Marangon S. The Avian influenza epidemic in Italy, 1999-−2000: a review. Avian Pathol. (2000) 29:289–94. 10.1080/03079450050118403 19184817

[17] Ravi N Cortade DL Ng E Wang SX. Diagnostics for SARS-CoV-2 detection: a comprehensive review of the FDA-EUA COVID-19 testing landscape. Biosens Bioelectron. (2020) 165:112454. 10.1016/j.bios.2020.112454 32729549 PMC7368663

[18] Jarrah SA Kmetiuk LB Valleriani F Bonfini B Lorusso A Vasinioti V. SARS-CoV-2 antibodies in dogs and cats in a highly infected area of Brazil during the pandemic. Front Vet Sci. (2023) 10:1111728. 10.3389/fvets.2023.1111728 36908526 PMC9995883

[19] Woelk CH Holmes EC. Reduced positive selection in vector-borne RNA viruses. Mol Biol Evol. (2002) 19:2333–6. 10.1093/oxfordjournals.molbev.a004059 12446826

[20] Pormohammad A Zarei M Ghorbani S Mohammadi M Razizadeh MH Turner DL. Efficacy RJ, and safety of COVID-19 vaccines: a systematic review and meta-analysis of randomized clinical trials. Vaccines. (2021) 9:467. 10.3390/vaccines9050467 34066475 PMC8148145

[21] Wu R Wang L Kuo HCD Shannar A Peter R Chou PJ. an update on current therapeutic drugs treating COVID-19. Curr Pharmacol Rep. (2020) 6:56–70. 10.1007/s40495-020-00216-7 32395418 PMC7211915

[22] Siddiquei MI Khan W. Economic implications of coronavirus. J Public Aff. (2020) 20:e2169. 10.1002/pa.2169 32837314 PMC7323339

[23] Gössling S Scott D Hall CM. Pandemics tourism and global change: a rapid assessment of COVID-19. J Sustain Tour. (2020) 29:1–20. 10.1080/09669582.2020.1758708

[24] Meltzer MI Cox NJ Fukuda K. The economic impact of pandemic influenza in the united states: priorities for intervention. Emerg Infect Dis. (1999) 5:659. 10.3201/eid0505.990507 10511522 PMC2627723

[25] Domingo E Holland J RNA. Virus mutations and fitness for survival. Annu Rev Microbiol. (1997) 51:151–78. 10.1146/annurev.micro.51.1.151 9343347

[26] Harfouch RM Moualla YM. Epidemiology of COVID-19 in the most pandemic countries: a review article. Acad J Biotechnol. (2021) 9:21–7.

[27] Willows S Hou S Hobman TC RNA. Virus capsid proteins: more than just a shell. Future Virol. (2013) 8:435–50. 10.2217/fvl.13.32

[28] Wulan WN Heydet D Walker EJ Gahan ME Ghildyal R. Nucleocytoplasmic transport of nucleocapsid proteins of enveloped RNA viruses. Front Microbiol. (2015) 6:553. 10.3389/fmicb.2015.00553 26082769 PMC4451415

[29] McDonald SM. RNA synthetic mechanisms employed by diverse families of RNA viruses. Wiley Interdiscip Rev RNA. (2013) 4:351–67. 10.1002/wrna.1164 23606593 PMC7169773

[30] Duffy S. Why Are RNA virus mutation rates so damn high? PLoS Biol. (2018) 16:e3000003. 10.1371/journal.pbio.3000003 30102691 PMC6107253

[31] V'kovski P Kratzel A Steiner S Stalder H Thiel V. Coronavirus biology and replication: implications for SARS-CoV-2. Nat Rev Microbiol. (2021) 19:155–70. 10.1038/s41579-020-00468-6 33116300 PMC7592455

[32] Strauss EG Strauss JH. Replication Strategies of the Single Stranded RNA Viruses of Eukaryotes. Cham: Springer. (1983). 10.1007/978-3-642-69159-1_1 6354610

[33] Sidorenko Y Reichl U. Structured model of influenza virus replication in MDCK cells. Biotechnol Bioeng. (2004) 88:1–14. 10.1002/bit.20096 15384040

[34] Holland J Spindler K Horodyski F Grabau E Nichol S. VandePol S. Rapid evolution of RNA genomes. Science. (1982) 215:1577–85. 10.1126/science.7041255 7041255

[35] Denison MR Graham RL Donaldson EF Eckerle LD Baric RS. Coronaviruses: an RNA proofreading machine regulates replication fidelity and diversity. RNA Biol. (2011) 8:270–9. 10.4161/rna.8.2.15013 21593585 PMC3127101

[36] Combe M Sanjuán R. Variability in the mutation rates of RNA viruses. Future Virol. (2014) 9:605–15. 10.2217/fvl.14.41

[37] La Rosa G Fratini M Libera SD Iaconelli M Muscillo M. Viral infections acquired indoors through airborne, droplet or contact transmission. Ann Ist Super Sanita. (2013) 49:124–32. 10.4415/ANN_13_02_03 23771256

[38] Judson S Prescott J Munster V. Understanding ebola virus transmission. Viruses. (2015) 7:511–21. 10.3390/v7020511 25654239 PMC4353901

[39] Moya A Holmes EC González-Candelas F. The population genetics and evolutionary epidemiology of RNA viruses. Nat Rev Microbiol. (2004) 2:279–88. 10.1038/nrmicro863 15031727 PMC7096949

[40] Simancas-Racines A Cadena-Ullauri S Guevara-Ramírez P Zambrano AK Simancas-Racines D. Avian influenza: strategies to manage an outbreak. Pathogens. (2023) 12:610. 10.3390/pathogens12040610 37111496 PMC10145843

[41] Ghadimipour R Taghizadeh M Bashashati M Ebrahimi MM Samadi A Mohammadzadeh S. Monitoring of newcastle disease virus vaccine strain replication in embryonated chicken eggs by reverse transcription-polymerase chain reaction. Arch Razi Inst. (2023) 78:807–13. 10.22092/ARI.2022.359142.2377 37396741 PMC10314241

[42] Oh D De Spiegelaere W Nauwynck H. Selection and validation of reference genes for RT-qPCR normalization of porcine alveolar macrophages (PAMs) for PRRSV studies. Sci Rep. (2023) 13:8840. 10.1038/s41598-023-35873-3 37258711 PMC10232543

[43] Alotaibi BS Tantry BA Bandy A Ahmad R Khursheed SQ Ahmad A. Simultaneous detection of influenza A/B, respiratory syncytial virus, and SARS-CoV-2 in nasopharyngeal swabs by one-tube multiplex reverse transcription polymerase chain reaction. Trop Med Infect Dis. (2023) 8:326. 10.3390/tropicalmed8060326 37368744 PMC10305207

[44] Shaib H Hussaini H Sleiman R Iskandarani Y Obeid Y. Development and validation of an indirect whole-virus ELISA using a predominant genotype VI velogenic newcastle disease virus isolated from lebanese poultry. Open J Vet Med. (2023) 13:82–95. 10.4236/ojvm.2023.136008

[45] Luo S Deng X Xie Z Huang J Zhang M Li M. Production and identification of monoclonal antibodies and development of a sandwich ELISA for detection of the H3-subtype avian influenza virus antigen. AMB Express. (2020) 10:1–9. 10.1186/s13568-020-00988-7 32170425 PMC7070111

[46] Baborenko YP Byadovskaya O Biryuchenkov D Konstantinov A Yashin R. Development and use of indirect liquid-phase ELISA test system for detection of PRRS virus antigen during in-process control of raw materials intended for vaccine production. Vet Sci Today. (2020) 33:109–114. 10.29326/2304-196X-2020-2-33-109-114

[47] Zhang Y Xu G Zhang L Zhao J Ji P Li Y. Development of a double monoclonal antibody–based sandwich enzyme-linked immunosorbent assay for detecting canine distemper virus. Appl Microbiol Biotechnol. (2020) 104:10725–35. 10.1007/s00253-020-10997-y 33159543 PMC7671975

[48] Taesuji M Rattanamas K Kulthonggate U Mamom T Ruenphet S. Sensitivity and specificity for african horse sickness antibodies detection using monovalent and polyvalent vaccine antigen-based dot blotting. Vet World. (2022) 15:2760. 10.14202/vetworld.2022.2760-2763 36718334 PMC9880840

[49] Brant AC Tian W Majerciak V Yang W Zheng ZM. SARS-CoV-2: From Its discovery to genome structure, transcription, and replication. Cell Biosci. (2021) 11:1–17. 10.1186/s13578-021-00643-z 34281608 PMC8287290

[50] Spaan W Cavanagh D. Horzinek, coronaviruses: structure m, genome expression. J Gen Virol. (1988) 69:2939–52. 10.1099/0022-1317-69-12-2939 3058868

[51] Satarker S. Nampoothiri M. Structural proteins in severe acute respiratory syndrome coronavirus-2. Arch Med Res. (2020) 51:482–91. 10.1016/j.arcmed.2020.05.012 32493627 PMC7247499

[52] Carabelli AM Peacock TP Thorne LG Harvey WT Hughes J Peacock SJ. SARS-CoV-2 variant biology: immune escape, transmission and fitness. Nat Rev Microbiol. (2023) 21:162–77. 10.1038/s41579-022-00841-7 36653446 PMC9847462

[53] Telenti A Hodcroft EB Robertson DL. The evolution and biology of SARS-CoV-2 variants. Cold Spring Harb Perspect Med. (2022) 12:a041390. 10.1101/cshperspect.a041390 35444005 PMC9159258

[54] Willett BJ Grove J MacLean OA Wilkie C Logan N Lorenzo GD. The hyper-transmissible SARS-CoV-2 omicron variant exhibits significant antigenic change, vaccine escape and a switch in cell entry mechanism. MedRxiv (2022) 2022:01. 10.1101/2022.01.03.21268111

[55] Tiwari R Dhama K Sharun K Iqbal Yatoo M Malik YS Singh R. COVID-19: Animals veterinary and zoonotic links. Vet Q. (2020) 40:169–82. 10.1080/01652176.2020.1766725 32393111 PMC7755411

[56] Mishra J Mishra P Arora NK. Linkages between environmental issues and zoonotic diseases: with reference to COVID-19 pandemic. Environ Sustain. (2021) 4:455–67. 10.1007/s42398-021-00165-x PMC8005368 38624661

[57] Ulrich L Wernike K Hoffmann D Mettenleiter TC Beer M. Experimental infection of cattle with SARS-CoV-2. Emerg Infect Dis. (2020) 26:2979. 10.3201/eid2612.203799 33034284 PMC7706945

[58] Albina E. Epidemiology of porcine reproductive and respiratory syndrome (PRRS): an overview. Vet Microbiol. (1997) 55:309–16. 10.1016/S0378-1135(96)01322-3 9220627

[59] Mötz M Stadler J Kreutzmann H Ladinig A Lamp B Auer A. Conserved stem-loop structure within ORF5 is a frequent recombination hotspot for porcine reproductive and respiratory syndrome virus 1 (PRRSV-1) with a particular modified live virus (MLV) strain. Viruses. (2023) 15:258. 10.3390/v15010258 36680298 PMC9867337

[60] Music N Gagnon CA. The role of porcine reproductive and respiratory syndrome (PRRS) virus structural and non-structural proteins in virus pathogenesis. Anim Health Res Rev. (2010) 11:135–63. 10.1017/S1466252310000034 20388230

[61] Wissink E Kroese M Van Wijk H Rijsewijk F Meulenberg J Rottier P. Envelope protein requirements for the assembly of infectious virions of porcine reproductive and respiratory syndrome virus. J Virol. (2005) 79:12495–506. 10.1128/JVI.79.19.12495-12506.2005 16160177 PMC1211556

[62] Kappes MA Faaberg KS PRRSV. structure replication and recombination: origin of phenotype and genotype diversity. Virology. (2015) 479:475–86. 10.1016/j.virol.2015.02.012 25759097 PMC7111637

[63] Stadejek T Stankevicius A Murtaugh MP Oleksiewicz MB. Molecular evolution of PRRSV in Europe: current state of play. Vet Microbiol. (2013) 165:21–8. 10.1016/j.vetmic.2013.02.029 23528651

[64] Lunney JK Fang Y Ladinig A Chen N Li Y Rowland B. Porcine reproductive and respiratory syndrome virus (PRRSV): pathogenesis and interaction with the immune system. Annu Rev Anim Biosci. (2016) 4:129–54. 10.1146/annurev-animal-022114-111025 26646630

[65] Franzo G Barbierato G Pesente P Legnardi M Tucciarone CM Sandri G. Porcine reproductive and respiratory syndrome (PRRS) epidemiology in an integrated pig company of northern italy: a multilevel threat requiring multilevel interventions. Viruses. (2021) 13:2510. 10.3390/v13122510 34960778 PMC8705972

[66] Madapong A Saeng-Chuto K Boonsoongnern A Tantituvanont A Nilubol D. Cell-Mediated immune response and protective efficacy of porcine reproductive and respiratory syndrome virus modified-live vaccines against co-challenge with PRRSV-1 and PRRSV-2. Sci Rep. (2020) 10:1649. 10.1038/s41598-020-58626-y 32015495 PMC6997162

[67] Peiris JM De Jong MD Guan Y. Avian influenza virus (H5N1): a threat to human health. Clin Microbiol Rev. (2007) 20:243–67. 10.1128/CMR.00037-06 17428885 PMC1865597

[68] Skelton RM Huber VC. Comparing influenza virus biology for understanding influenza d virus. Viruses. (2022) 14:1036. 10.3390/v14051036 35632777 PMC9147167

[69] Asha K Kumar B. Emerging influenza D virus threat: what we know so far! *J Clin Med*. (2019) 8:192. 10.3390/jcm8020192 30764577 PMC6406440

[70] Liu R Sheng Z Huang C Wang D Li F. Influenza D virus. Curr Opin Virol. (2020) 44:154–61. 10.1016/j.coviro.2020.08.004 32932215 PMC7755673

[71] Zhu Z Fodor E Keown JR. A structural understanding of influenza virus genome replication. Trends Microbiol. (2022). 10.1016/j.tim.2022.09.015 36336541

[72] Haddas R. Newcastle disease virus. Infect Dis. (2023) 2023:427. 10.1007/978-1-0716-2463-0_1093

[73] Haenni AL Joshi S Chapeville F. RNA-like structures in the genomes of RNA viruses. Prog Nucleic Acid Res Mol Biol. (1982) 27:85–104. 10.1016/S0079-6603(08)60598-X 6285419

[74] Peeters B Gruijthuijsen Y De Leeuw O Gielkens A. Genome replication of newcastle disease virus: involvement of the rule-of-six. Arch Virol. (2000) 145:1829–45. 10.1007/s007050070059 11043944

[75] Vandevelde M. The pathogenesis of nervous distemper. Vet Sci Tomorrow. (2005) 127:1–18.

[76] Martinez-Gutierrez M Ruiz-Saenz J. Diversity of susceptible hosts in canine distemper virus infection: a systematic review and data synthesis. BMC Vet Res. (2016) 12:1–11. 10.1186/s12917-016-0702-z 27170307 PMC4865023

[77] Carvalho OV Botelho CV Ferreira CGT Scherer PO Soares-Martins JAP Almeida MR. Immunopathogenic and neurological mechanisms of canine distemper virus. Adv Virol. (2012) 2012:163860. 10.1155/2012/163860 23193403 PMC3501799

[78] Nagai Y. Paramyxovirus replication and pathogenesis. Reverse genetics transforms understanding. Rev Med Virol. (1999) 9:83–99. 10386336 10.1002/(sici)1099-1654(199904/06)9:2<83::aid-rmv244>3.0.co;2-5

[79] Cleaveland S Kaare M Knobel D Laurenson MK. Canine vaccination—providing broader benefits for disease control. Vet Microbiol. (2006) 117:43–50. 10.1016/j.vetmic.2006.04.009 16701966

[80] Schultz RD. Duration of immunity for canine and feline vaccines: a review. Vet Microbiol. (2006) 117:75–9. 10.1016/j.vetmic.2006.04.013 16707236

[81] Zientara S Weyer CT Lecollinet S. African horse sickness. Rev Sci Tech. (2015) 34:315–27. 10.20506/rst.34.2.2359 26601437

[82] Dennis SJ Meyers AE Hitzeroth II Rybicki EP. African Horse Sickness: A Review of Current Understanding and Vaccine Development. Viruses. (2019) 11:844. 10.3390/v11090844 31514299 PMC6783979

[83] Roy P Mertens PP Casal I. African horse sickness virus structure. Comp Immunol Microbiol Infect Dis. (1994) 17:243–73. 10.1016/0147-9571(94)90046-9 8001348

